# Efficacy of immunotherapy in *ARID1A*-mutant solid tumors: a single-center retrospective study

**DOI:** 10.1007/s12672-024-01074-1

**Published:** 2024-06-07

**Authors:** Hai Zhou, Dantong Sun, Shanai Song, Yurong Niu, Yuming Zhang, Hongwei Lan, Jiali Cui, Houde Liu, Ning Liu, Helei Hou

**Affiliations:** 1 Department of Oncology, The Affiliated Hospital of Qingdao University, No. 7 Jiaxing Road, Qingdao, 266000 Shandong China; 2 Department of Medical Oncology, National Cancer Center/National Clinical Research Center for Cancer/Cancer Hospital, Chinese Academy of Medical Sciences and Peking Union Medical College, Beijing, 100021 China; 3 Precision Medicine Center of Oncology, The Affiliated Hospital of Qingdao University, Qingdao, 266000 Shandong China; 4 Medical College of Qingdao University, No.308 Ningxia Road, Qingdao, 266000 Shandong China

**Keywords:** *ARID1A*, Next-generation sequencing, Biomarker, Precision medicine, Tumor immune microenvironment

## Abstract

**Background:**

Immune checkpoint inhibitors (ICIs), especially those targeting programmed cell death-1 (PD-1) and programmed cell death ligand-1 (PD-L1), have introduced a new treatment landscape for many types of tumors. However, they only achieve a limited therapeutic response. Hence, identifying patients who may benefit from ICIs is currently a challenge.

**Methods:**

47 tumor patients harboring *ARID1A* mutations were retrospectively studied. The genomic profiling data through next-generation sequencing (NGS) and relevant clinical information were collected and analyzed. Additionally, bioinformatics analysis of the expression of immune checkpoints and immune cell infiltration levels was conducted in *ARID1A*-mutant gastric cancer (GC).

**Results:**

*ARID1A* mutations frequently co-occur with mutations in DNA damage repair (DDR)-associated genes. Among the 35 *ARID1A*-mutant patients who received immunotherapy, 27 were evaluable., with the objective response rate (ORR) was 48.15% (13/27), and the disease control rate (DCR) was 92.59% (25/27). Moreover, survival assays revealed that *ARID1A*-mutant patients had longer median overall survival (mOS) after immunotherapy. In *ARID1A*-mutated GC patients, receiving ICIs treatment indicated longer progressive-free survival (PFS). Additionally, the incidence of microsatellite instability-high (MSI-H), high tumor mutation burden (TMB-H) and Epstein‒Barr virus (EBV) infection was elevated. Bioinformatic analysis showed significant enrichment of immune response and T cell activation pathway within differentially expressed genes in *ARID1A*-mutant GC group. Finally, *ARID1A* mutations status was considered to be highly correlated with the level of tumor infiltrating lymphocytes (TILs) and high expression of immune checkpoints.

**Conclusions:**

Patients with tumors harboring *ARID1A* mutations may achieve better clinical outcomes from immunotherapy, especially in GC. *ARID1A* mutations can lead to genomic instability and reshape the tumor immune microenvironment (TIME), which can be used as a biomarker for immunotherapy.

**Supplementary Information:**

The online version contains supplementary material available at 10.1007/s12672-024-01074-1.

## Introduction

Immune checkpoint inhibitors (ICIs) have generated clinical efficacy across a wide array of tumor types, including mismatch repair-deficient and microsatellite instability-high (d-MMR/MSI-H) cancers [1, 2]. However, despite the success of ICIs, the clinical responses varies among patients [3], with only 20–40% of patients benefiting from these revolutionary therapies [4, 5]. Therefore, exploring novel predictive biomarkers is critical.

AT-rich interaction domain 1A (*ARID1A*), a gene encoding a large nuclear protein member of the switch/sucrose non-fermentation (SWI/SNF) chromatin remodeling complex, may downregulate corresponding protein levels due to the functional mutation [6]. As a known tumor suppressor gene, *ARID1A* strongly regulates the DNA repair pathway, thus driving tumor formation [7]. In our previous study, ARID1A was found to serve as a novel biomarker for the prognosis and sensitivity to ICIs of advanced non-small cell lung cancer (NSCLC) [8, 9]. This finding indicates that the *ARID1A* mutation status has potential predictive value in immunotherapy.

In this study, we retrospectively analyzed the genomic alterations and clinical outcomes of patients harboring *ARID1A* mutations. Then, the predicted functions of *ARID1A* mutations in gastric cancer (GC) were comprehensively analyzed.

## Materials and methods

### Study population

57 tumor patients from the Affiliated Hospital of Qingdao University were retrospectively studied, including 47 patients with *ARID1A* mutations and 10 *ARID1A* wild-type GC patients undergoing immunotherapy. Each patient’s baseline information, clinical efficacy and follow-up information were collected. The clinical characteristics are shown in Supplementary Table 1. Treatment response to immunotherapy was evaluated based on the Response Evaluation Criteria in Solid Tumors (RECIST) version 1.1. Objective response rate (ORR) was calculated as complete response (CR) rate plus partial response (PR) rate under computed tomography or magnetic resonance imaging. Disease control tare (DCR) was defined as proportion of patients who had a CR, PR, and stable disease (SD) as best overall response during immunotherapy. All procedures were approved by the Ethics Committee of the Affiliated Hospital of Qingdao University. All investigations were carried out according to the rules of the Declaration of Helsinki.

### Next-generation sequencing assay

Next-generation sequencing (NGS) assays from Burning Rock Co. and BGI Co. were used to analyze tissue samples. Probe hybridization and high-throughput sequencing were employed to detect the whole-exon region of 310 genes and the hot spot mutation region (including exon, intron or promoter region) of 210 genes. The assay covers single nucleotide variants (SNVs) in the target gene capture exons and short fragment insertion or deletion variants (Indels), copy number variants (CNVs), and gene rearrangements (Rearrangements/Fusions) with breakpoints within the capture range. In tissue specimen mutation detection, using 1% as the minimum threshold for determining the variant allele frequency (VAF) of a mutation. Our analysis focused on genetic mutations in SWI/SNF complex members, including ARID and SMARC family genes, DNA damage response (DDR) genes, immune-related genes, and common cancer driver genes. All variants of unknown significance (VUS) were excluded from further studies, including genes that have not yet approved by clinical practice guidelines or lack clinical evidence. We examined five microsatellite loci (BAT25, BAT26, D17S250, D2S123 and D5S346) from patients’ tumor tissue and their matched blood as controls, with 2 or more out of 5 loci mutations regarded as MSI-H, 1 and none mutated loci as microsatellite instability-low (MSI-L)/microsatellite stability (MSS).

### Database and bioinformatics analysis

The cBioPortal (https://www.cbioportal.org) is a collection of multidimensional cancer genomics datasets [10, 11]. Therefore, *ARID1A* alterations were analyzed and visualized in GC studies and immunogenomic cohorts from the cBioPortal database [12–14].

MuTarget (https://www.mutarget.com/), which is a target discovery tool that connects mutation status to gene expression changes in solid tumors, was used to identify differentially expressed genes (DEGs) between *ARID1A*-mutant (MUT) and *ARID1A*-wild type (WT) GC patients [15]. Default thresholds of *P* < 0.01 and fold change (FC) > 1.44 were used to identify DEGs.

DAVID (https://david.ncifcrf.gov/home.jsp), a website tool that provides a comprehensive functional annotation of a group of genes [16], was used to perform Gene Ontology (GO) and Kyoto Encyclopedia of Genes and Genomes (KEGG) enrichment analyses and DisGeNET analysis of DEGs. *P* < 0.05 was considered statistically significant.

The TISIDB database (http://cis.hku.hk/TISIDB/index.php) were used to explore the association between *ARID1A* mutations and microenvironment components [17].

### Statistical analysis

Overall survival (OS) was calculated as the time from diagnosis to death. Progressive-free survival (PFS) was referred to time from immunotherapy initiation until disease progression (PD) or mortality of any cause. OS and PFS were plotted by the Kaplan‒Meier method. The curve was compared by using the log-rank test. *P*-values were determined by two-tailed tests. All P-values of less than 0.05 were considered significant. Statistical analyses were performed using SPSS Statistics v.25 software.

## Results

### Clinical characteristics of *ARID1A*-mutant tumor patients

In our cohort, 33 patients received immunotherapy, of whom 16 had gastric cancer, 4 had endometrial cancer, 3 had non-small cell lung cancer, 3 had cholangiocarcinoma, and 2 had esophageal carcinoma. The remaining 5 patients had urothelial cancer, ovarian cancer, colon cancer, breast cancer, and pancreatic cancer, respectively. In terms of gender, 21 were males, and 12 were females. The median age was 59.5 years (range 40–78 years). 21 patients (21/33, 63.64%) at an advanced stage, 10 patients were stage III, and 2 patients were stage II. The detailed clinical features of all enrolled patients are summarized in Table 1.

**Table 1:** Characteristics of *ARID1A*-mutant patients receiving anti-PD-1/PD-L1 immunotherapy (n = 33)

Characteristics	No of cases (%)
Sex	
Male	21 (63.64)
Female	12 (36.36)
Age	
< 60	20 (60.61)
≥ 60	13 (39.39)
Smoking history	
Yes	14 (42.42)
No	19 (57.58)
Histology	
Non-small cell lung cancer	3 (9.09)
Gastric cancer	16 (48.49)
Endometrial cancer	4 (12.12)
Ureteral cancer	1 (3.03)
Cholangiocarcinoma	3 (9.09)
Ovarian cancer	1 (3.03)
Esophageal cancer	2 (6.06)
Colorectal cancer	1 (7.69)
Breast cancer	1 (3.039)
Pancreatic cancer	1 (3.03)
Stage	
I	0 (0)
II	2 (6.06)
III	10 (30.30)
IV	21 (63.64)
PD-L1 expression (CPS)	
< 1	7 (21.21)
≥ 1	8 (24.24)
≥ 10	12 (36.37)
Unknow	6 (18.18)
TMB	
< 10	15 (45.45)
≥ 10	18 (54.55)
MSI	
MSS	19 (57.58)
MSI-H	11 (33.33)
Unknow	3 (9.09)
Lines of immunotherapy	
Neoadjuvant	3 (9.09)
1	19 (57.58)
2	5 (15.15)
≥ 3	6 (18.18)
Therapy responds	
CR	2 (6.06)
PR	11 (33.33)
PD	3 (9.09)
SD	12 (36.37)
NA	5 (15.15)

*TMB* Tumor mutation burden, *MSS* Microsatellite stability, *MSI-H* Microsatellite instability-high, *CR* Complete response, *NA* Not applicable, *PD* Progressive disease, *PD-L1* Programmed death-ligand 1, *PR* Partial response, *SD* Stable disease, *PD* Progress disease

### PD-L1 expression, TMB, MSI status and NGS assay

Among the entire cohort, PD-L1 expression was TPS ≥ 1% in 18 patients (18/47, 38.30%). In addition, PD-L1 expression was negative (TPS < 1%) in 9 patients. In 9 cases, the CPS ranged from 1 to 10, while in 17 cases, the CPS was ≥ 10. TMB was evaluated in all patients, of whom 23 patients (23/47, 48.94%) had TMB ≥ 10, and the median TMB was 42.36 muts/MB (range 1.08–233 muts/MB). 13 patients (13/47, 27.66%) demonstrated MSI-H tumors (Table 1).

**Fig. 1:**
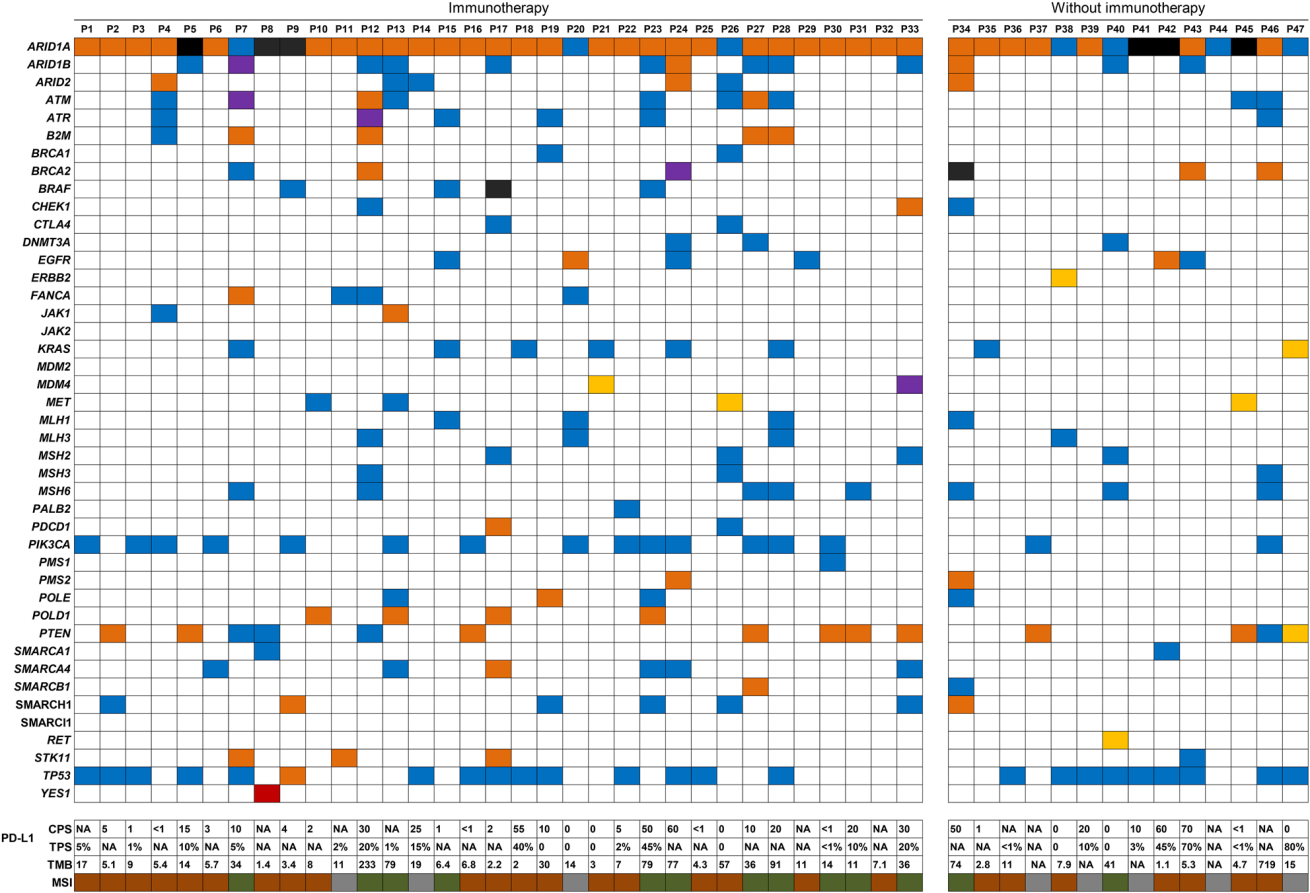
Molecular characteristics of patients with *ARID1A* mutations. Blue boxes indicate missense mutations, orange boxes are truncating mutations, yellow boxes present copy number gain, purple boxes are splice mutations, black boxes are deep deletion, red boxes are fusion, and green boxes are MSI-H, brown boxes are MSS/MSI-L, and gray boxes present unknow. *NA* Not applicable, *MSI* Microsatellite instability, *MSS* Microsatellite stability, *PD-L1* Programmed cell death ligand-1

The NGS analysis of all patients was summarized in Fig. 1. 24 of 47 patients (24/47, 51.06%) had *TP53* mutations. *PTEN* mutations were identified in 14 patients (14/47, 29.78%). *ARID1B, SMARCA4,* and *SMARCH1,* the SWI/SNF family genes, accounted for 27.66%, 12.77%, and 14.89% of the mutations, respectively. Additionally, 15 patients (15/47, 29.79%) had at least one genetic mutation related to the MMR pathway, including *MLH1, MLH3, MSH2, MSH3, MSH6, PMS1,* and *PMS2*. The detailed genetic mutation information can be found in Supplemental Table 1.

### Efficacy of immunotherapy in patients with *ARID1A* mutations

**Fig. 2:**
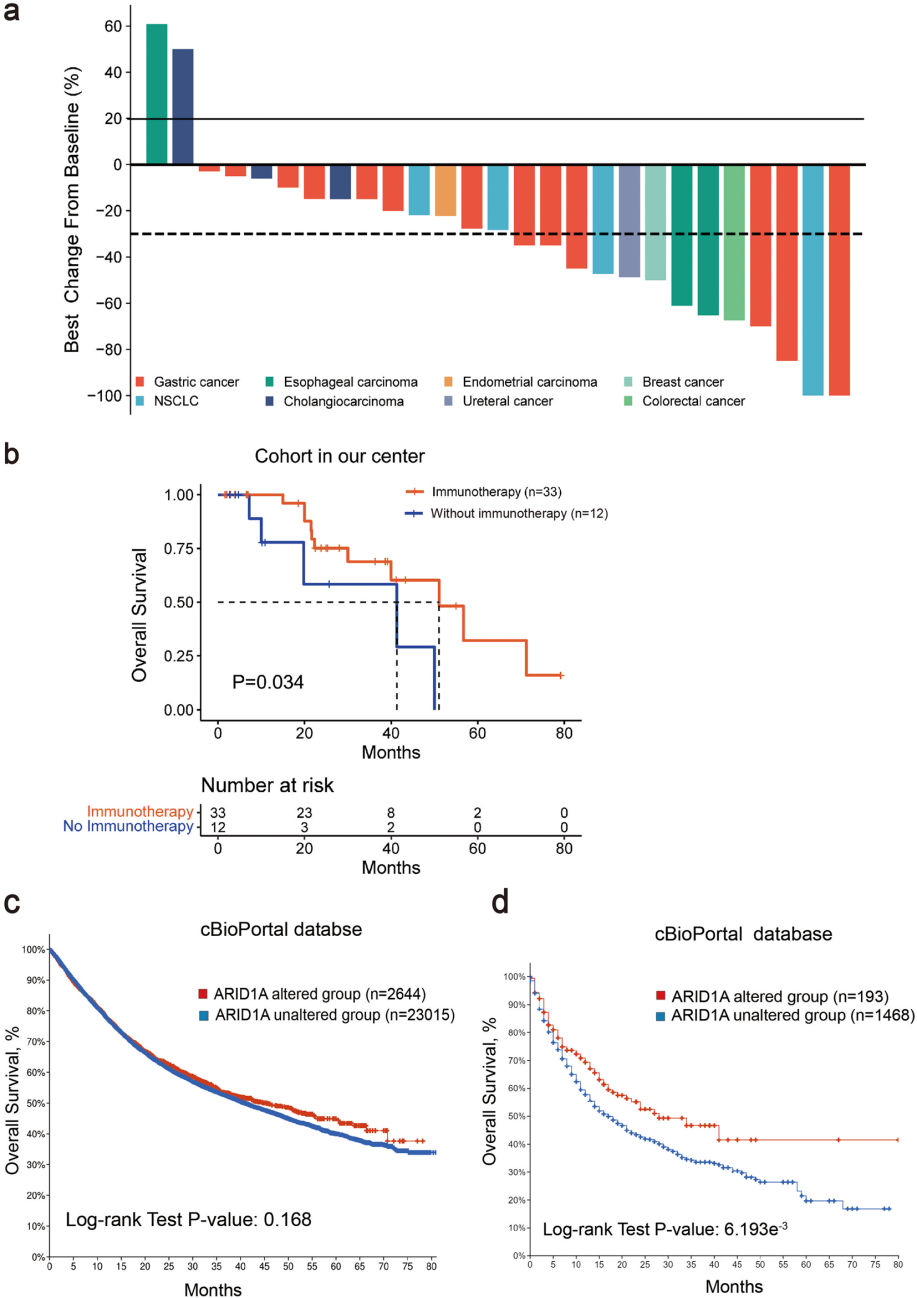
Patients harboring *ARID1A* mutations have better clinical outcomes after immunotherapy. **a** Waterfall plot of the maximum shrinkage rate of measurable targeted lesions compared with baseline **b**. Patients with *ARID1A* mutations who received immunotherapy exhibited longer OS in our cohort (P = 0.034). **c**. *ARID1A* mutations status was not correlated with OS in pan-cancer setting by cBioPortal database analysis **d**. OS among patients received immunotherapy with *ARID1A* mutations or not from the cBioPortal database. *OS* Overall survival

Among the 33 patients receiving ICIs treatment, 27 patients had assessable lesions were evaluable for response. A Waterfall plots were used to show the best observed changes in tumor size (Fig. 2a). The other 6 patients received maintenance therapy with ICIs after surgery, 1 of these patients had disease progression (PD) due to increased cancerous ascites, and none of the other 5 patients had an assessable lesion. Among the evaluable tumors, 13 patients (13/27, 48.15%) achieved a partial response (PR) and complete response (CR), and the DCR was 92.592% (25/27). Totally 7 of 27 were MSI-H, of whom 1 patients achieved a CR, 3 patient achieved a PR, 3 patients had a SD after immunotherapy.

Until the follow-up date, patients receiving immunotherapy achieved a mOS of 51.07 months. In contrast, patients without immunotherapy had a mOS of 41.33 months. Kaplan–Meier survival analysis revealed that the non-treated ICIs group had significantly shorter survival compared to the treated group (Hazard ratio (HR) = 0.346, 95% confidence interval (CI) 0.083–1.446, P = 0.034, Fig. 2b). Additionally, analysis using the cBioPortal database showed no significant difference in OS between *ARID1A*-altered patients and wild-type (WT) patients (Fig. 2c). However, in a pan-cancer immunotherapy cohort, patients with *ARID1A* alterations had significantly longer OS, with the difference being statistically significant (P = 6.193e^−3^) (Fig. 2d).

### Survival analysis of *ARID1A*-mutated malignant gastric cancer after immunotherapy

**Fig. 3:**
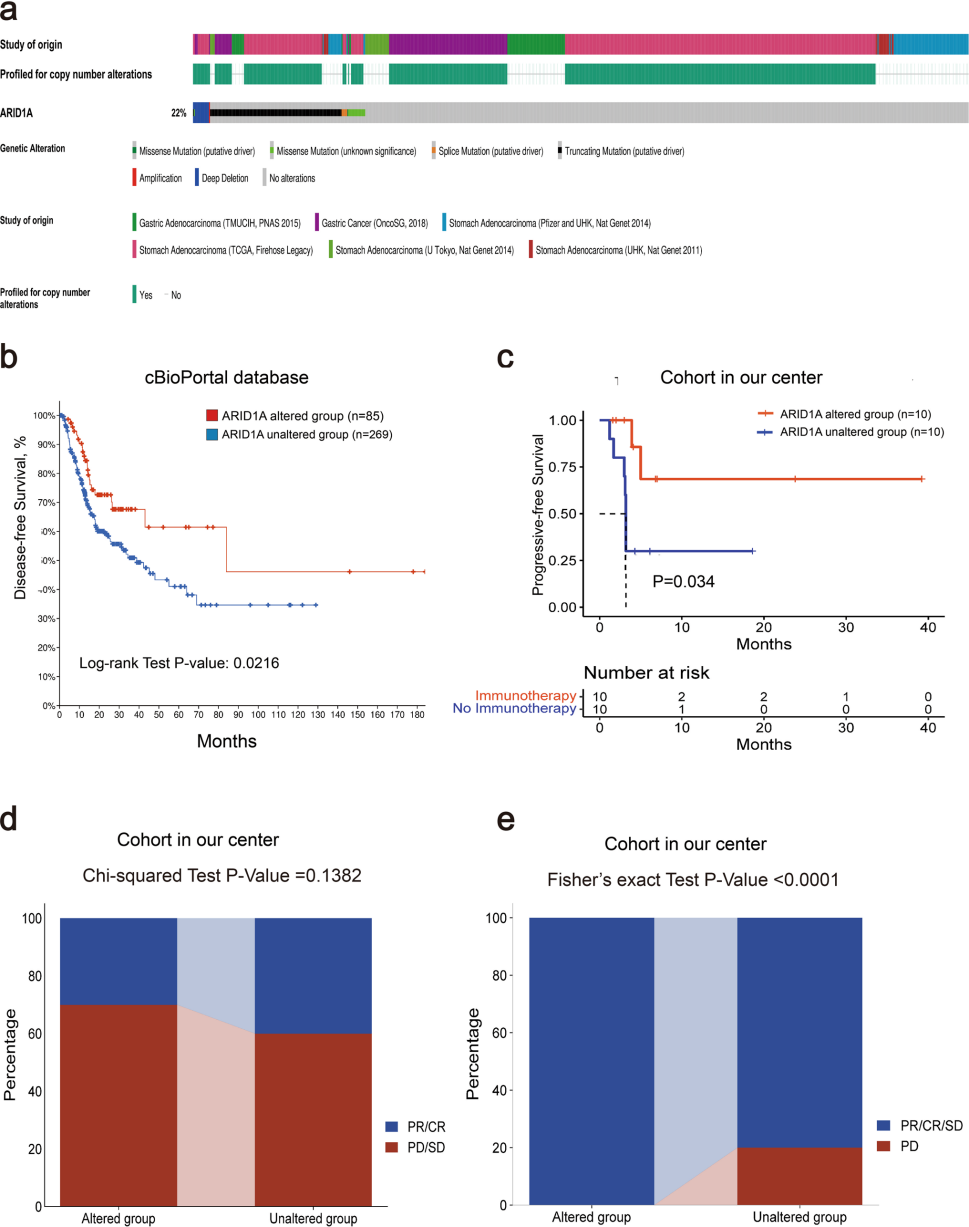
*ARID1A* mutations frequency and survival analysis of *ARID1A*-mutated GC. **a**. The incidence of *ARID1A* mutations in GC study cohorts from the cBioPortal database. **b**. GC patients with *ARID1A* mutations had longer DFS (P = 0.0216). **c**. Progressive-free survival of patients received immunotherapy with or without *ARID1A* mutations (P = 0.034). **d**–**e**. Proportion of patients responding to immunotherapy in our cohort. *GC* Gastric cancer, *DFS* Disease-free survival

We selected GC patients for further mechanistic analysis. We retrospectively gathered data of 10 *ARID1A*-WT GC patients who received first-line chemotherapy plus immunotherapy (Supplemental Fig. S1). In addition, 6 GC cohorts from the cBioPortal database were analyzed for cancer genomics. *ARID1A* mutations were detected in 22% (179/855) of GC patients, as shown in Fig. 3a, which indicated that *ARID1A* mutations was one of the most frequent gene mutations in GC. In terms of the alteration types, truncation mutations were the most common, followed by missense mutations. Survival analysis showed that GC patients with *ARID1A* mutations had a better prognosis, as demonstrated in Fig. 3b. The disease-free survival (DFS) of *ARID1A*-altered GC patients was significantly longer than that of *ARID1A*-WT patients (84.00 months vs. 38.90 months, P = 0.0216). Moreover, compared with *ARID1A*-mutated patients who underwent immunotherapy combined with chemotherapy as first-line treatment, *ARID1A* mutations was significantly associated with increased PFS (Not reached vs 3.2 months, HR = 0.225, 95% CI 0.061–0.841, P = 0.034, Fig. 3c).

In *ARID1A*-WT GC from our cohort, CR were observed in 0 patient, PR in 4 patients and SD in 4 patients, 2 patients had progressive disease (PD), resulting in an ORR of 40% and a DCR of 80%. In *ARID1A*-mutated group, 10 advanced GC patients received chemotherapy in combination with immunotherapy as a first-line treatment, of which 3 cases achieved (30.00%) CR/PR and 7 cases (70.00%) had SD. The ORR and DCR were 30 and 100%, respectively. The difference in ORR between two group was statistically insignificant (40 vs. 30%, respectively, P = 0.1382, Fig. 3d). However, the difference in DCR showed statistical significance (80 vs. 100%, respectively, P < 0.0001, Fig. 3e).

### The clinical attributes in *ARID1A*-mutated GC patients

**Fig. 4:**
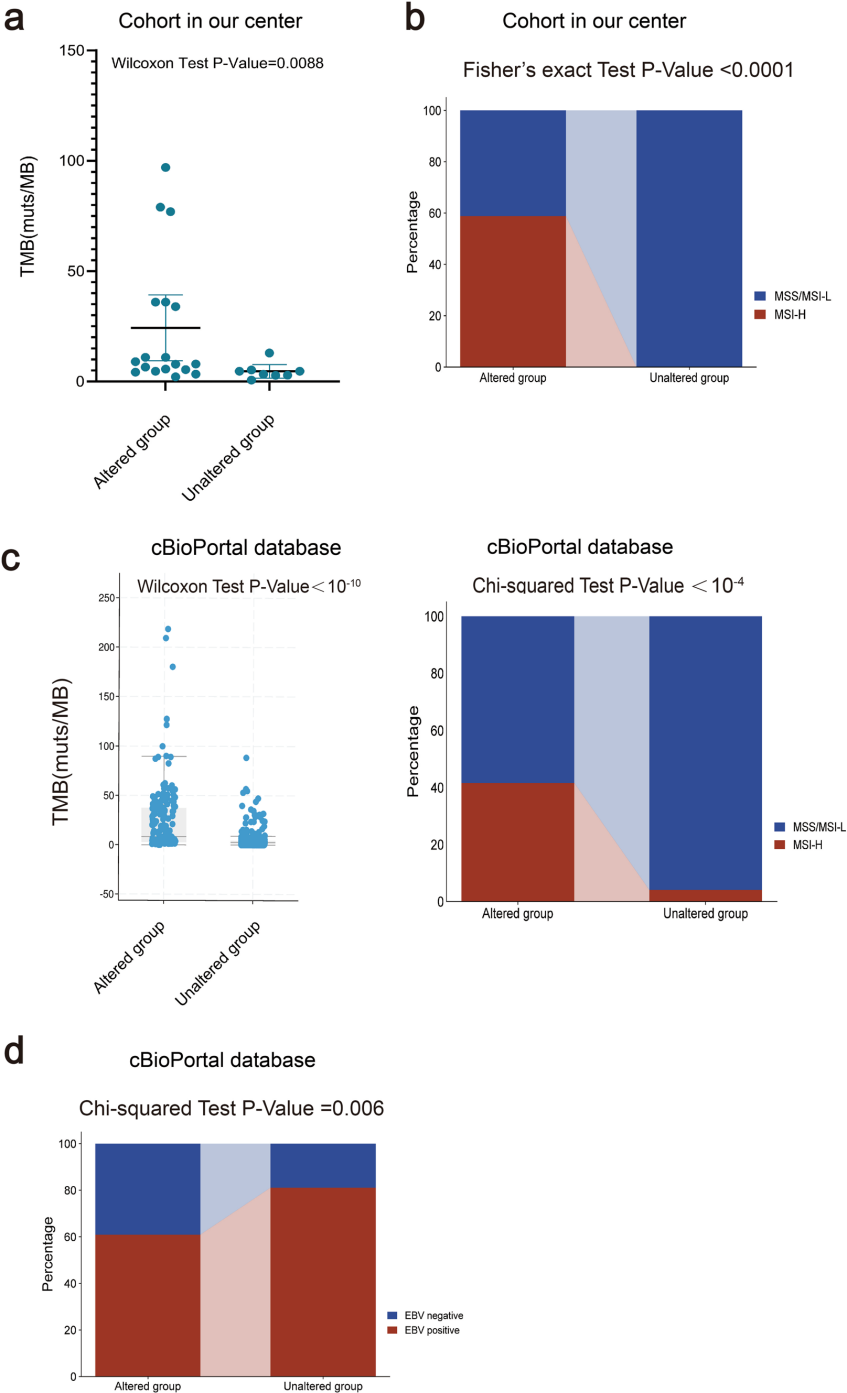
Comparison of the clinical attributes of the *ARID1A* wild-type and mutated GC groups. **a**. *ARID1A* mutations is associated with TMB level in GC of our cohort. **b**. The associated between MSI status and *ARID1A* mutations **c**. Comparison of TMB and MSI between *ARID1A* wild-type and mutated groups of the cBioPortal database. **d**. The correlation of EBV infection with *ARID1A* mutations. *GC* Gastric cancer, *TMB* Tumor mutation burden, *MSI* Microsatellite instability, *EBV* Epstein-Barr virus

We assessed the correlation between *ARID1A* status and clinical attributes in GC, considering the extensive influence on immunotherapy responsiveness. In our cohort, TMB value was significantly higher in GC patients with *ARID1A* mutations (Wilcoxon test P = 0.0088, Fig. 4a). In addition, more GC patients in the mutant group were identified with MSI-H, which suggested that these patients had more genomic instability (Fisher’s exact test P < 0.0001, Fig. 4b). The same results were corroborated in the cBioPortal database (Fig. 4c). Interestingly, the proportion of EBV-positive GC patients with *ARID1A* mutations was also higher than that of *ARID1A*-WT patients (Wilcoxon test P = 0.06, Fig. 4d).

### Correlations between *ARID1A* mutations and tumor immune microenvironment (TIME)

**Fig. 5:**
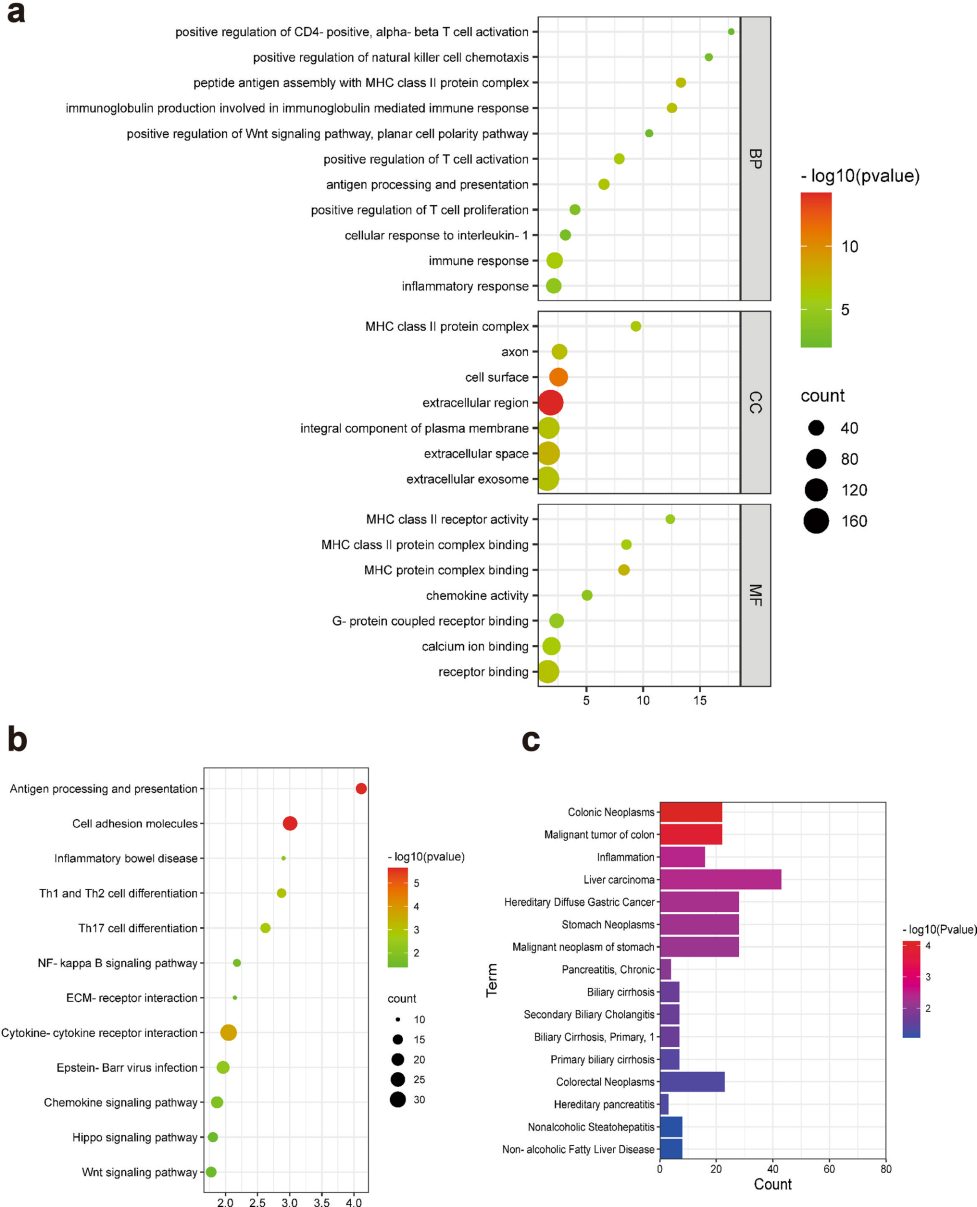
Functional enrichment analysis of DEGs. **a**–**c**. The results of enrichment analysis of the DEGs—namely, GO analysis, KEGG pathway analysis, and DisGeNET enrichment analysis. *DEGs* Differentially expressed genes

The relationship between *ARID1A* and TIME was further studied. A total of 904 DEGs, including 259 upregulated and 645 downregulated genes, were subjected to functional analysis via the DAVID website. GO term analysis revealed the accumulation of DEGs in T-lymphocyte activation, immune response, and inflammatory response. Regarding molecular function, DEGs were involved in receptor binding (Fig. 5a). KEGG pathway analysis showed that the DEGs were enriched in antigen processing and presentation pathways and cell adhesion molecules (Fig. 5b). According to the DisGeNET analysis, DEGs were found to be significantly enriched in colorectal tumors, liver cancer, stomach cancer, inflammation, etc. (Fig. 5c). Furthermore, the DEGs may be involved in Epstein‒Barr virus (EBV) infection, the Wnt signaling pathway, the chemokine signaling pathway and the Hippo signaling pathway.

**Fig. 6:**
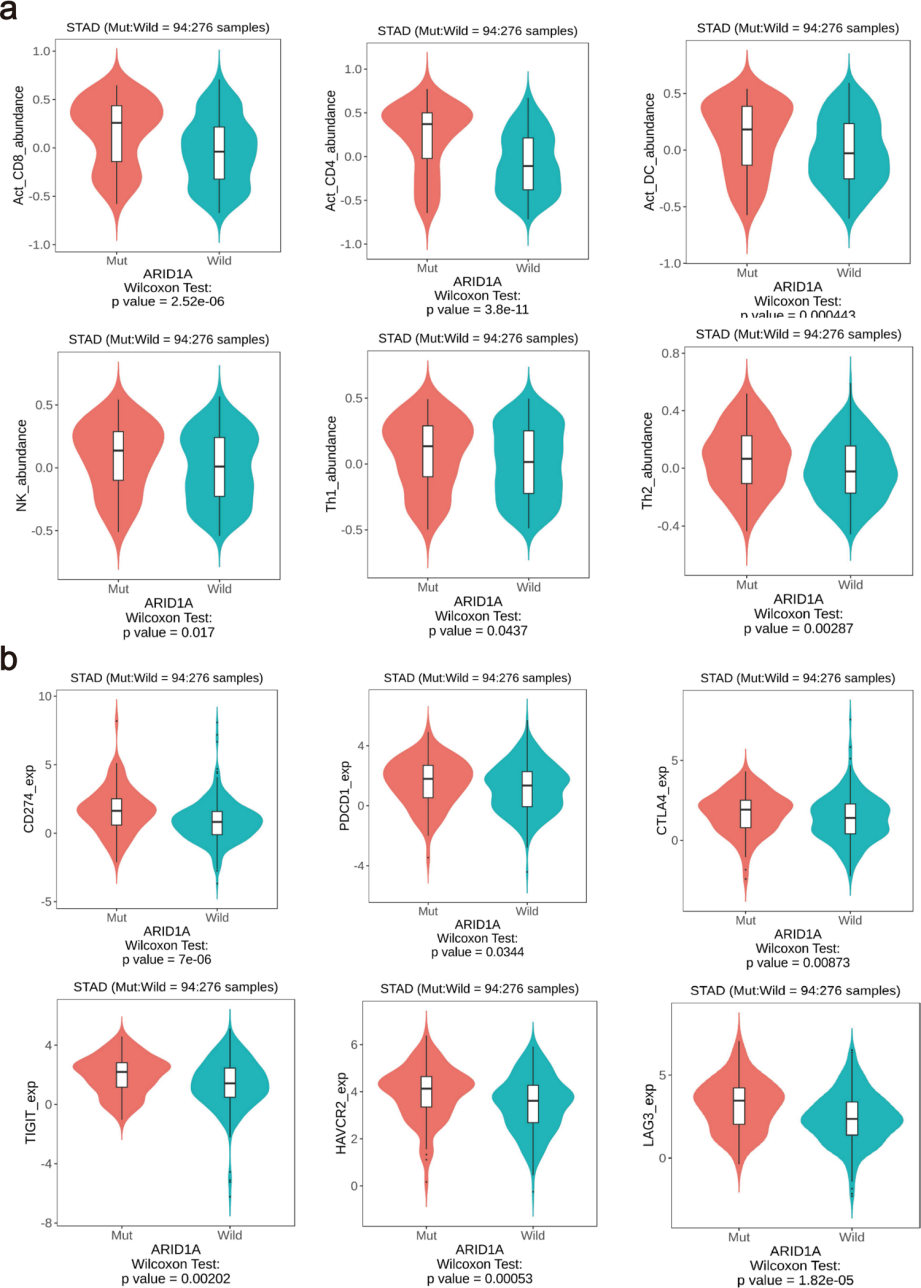
Correlations between *ARID1A* mutations and tumor immune environment in GC. **a** *ARID1A* mutations was highly associated with immune cells infiltration. **b**. The expression of immune checkpoint was influenced by *ARID1A* mutations status. *GC* Gastric cancer

Moreover, the correlation between the abundance of tumor-infiltrating lymphocytes (TILs) and *ARID1A* mutations status was analyzed in GC. Based on the TISIDB databases, we found a correlation between *ARID1A* mutations and the abundance of infiltrating immune cells, including activated CD8 + T cells (P = 2.52e^−6^), activated CD4 + T cells (P = 3.8e^−11^), activated dendritic cells (P = 0.000443), natural killer cells (P = 0.017), Th1 cells (P = 0.0437) and Th2 cells (P = 0.00287) (Fig. 6a). The association of *ARID1A* mutations with immune checkpoints was also analyzed. The results displayed that *ARID1A* mutations in GC was significantly associated with CD274 (P = 7e^−6^), PDCD1 (P = 0.0344), TIGIT (P = 0.00202), CTLA4 (P = 0.00873), HAVCR2 (P = 0.00053), and LAG3 (P = 1.82e^−5^) (Fig. 6b).

## Discussion

The failure of an adequate immune response to standard immunotherapy is a major clinical challenge [18]. Some elegant studies have described *ARID1A* mutations may be closely related to tumor immunity and immunotherapy [19–21]. However, most of these studies were basic research. A retrospective clinical study from real-world is lacking, and genomic features of the *ARID1A*-mutated patients remains unclear.

In our study, immunotherapy significantly prolonged the survival time of patients with *ARID1A* mutations. Among these patients, 2 patient achieved CR, and 11 patients achieved PR. All but PD patients had varying degrees of tumor shrinkage. Moreover, we validated our results on survival benefits in an independent immune cohort from a database. Thus, identifying potential immune therapeutic options based on *ARID1A* alterations is promising.

In line with other reports [22], the majority of *ARID1A* mutations in our study were inactivating mutations that could result in the loss of ARID1A expression. Moreover, in our cohort, *ARID1A* mutations are often accompanied by co-mutations in genes involved in DDR pathways, including *ATM*, *ATR*, *BRCA2*, etc. We found *MMR* pathway genes were frequently mutated, with *MSH6* mutations being the most common. These critical genes alterations decrease DNA repair capacity, therapy increasing tumor burden and activating immunity. Interestingly, in all patients, 48.94% patients had high TMB, and 27.66% patients were microsatellite instable. Consistent with the cBioPortal database analysis results, patients harboring *ARID1A* mutations were more likely to have increased genomic instability, while TMB levels were also significantly elevated. Currently, MSI-H/dMMR and TMB-H have already been recognized as positive indications for immunotherapy [23]. Thus, *ARID1A* mutations have potential application value for the prediction of response to ICIs. *Shen* et al. revealed that ARID1A inactivation hampered the recruitment of MSH2 to chromatin during DNA replication and induced dMMR in a proteomic screen [20]. In addition, deletions in ARID1A expression were associated with methylation of the promoter of the MLH1 gene [24]. These could be underlying mechanism. Furthermore, the DCR in ICIs-treated patients with MSS was 88.24% (15/17). This finding suggests that *ARID1A* mutations are still an effective predictor of ICI efficacy regardless of MSI status.

Previous researchers have revealed that EBV-positive tumors are related to checkpoint blockade responses [25, 26]. A possible factor is that EBV-positive cancers often exhibit amplification of the 9p24.1 locus linked to the overexpression of JAK2, CD274, and PDCD1LG2 [27]. Our findings showed that *ARID1A* mutations was associated with EBV infection in GC. This association implied potential benefits for ICIs therapy in some patients with *ARID1A* mutations in GC. Unfortunately, GC patients in our cohort were not tested for EBV status.

In pan-cancer patients, we investigated the correlation between *ARID1A* mutations and the immune cell microenvironment in GC. The latest literature [28] showed that, compared to HER2-positive GC patients, *ARID1A* mutations was significantly enriched in HER2-negative GC patients. HER2-negative GC with *ARID1A* mutations may be sensitive to ICIs, due to increased T-cell lymphocytosis. TILs play a vital role in the improved survival of cancers, and both the quantity and quality of TILs are possible factors in determining immune therapeutic benefits, especially T lymphocytes [29]. Moreover, NK cells were proven to be critical for the therapeutic effects of PD1 blockade [30]. Our findings confirmed that *ARID1A* mutations are associated with immune infiltration and immunosuppressive receptors in the TIME. GC patients harboring *ARID1A* alterations had a greater abundance of activated CD8 + /CD4 + T lymphocytes, DCs, and NK cells, which improved the sensitivity to ICIs. Furthermore, some immunosuppressive targets, such as PD-L1, CTLA-4, HAVCR2, LAG3, and TIGIT, had a significant expression difference in *ARID1A*-mutant GC. At present, accumulating studies have shown that HAVCR2 and LAG3 are valuable as potential targets for immunotherapy, and preclinical tumor models have shown that the use of immunoinhibitors to block HAVCR2, LAG3, and TIGIT restricts the growth of cancer masses [31–33]. Admittedly, our study has some limitations. This work is a single-center retrospective study, and multicenter prospective studies are needed to further verify the conclusions.

## Conclusions

Based on a single-center retrospective analysis, we concluded that *ARID1A* mutations were often co-mutated with DNA damage response genes and could be associated with better immunotherapy outcomes in solid tumors. Bioinformatics analysis confirmed the elevated expression of immunosuppressive receptors and increased immune cell infiltration in *ARID1A*-mutant tumors, both of which are beneficial to the use of ICIs and affect patient outcomes. Therefore, *ARID1A* can serve as a novel biomarker for immunotherapy for malignant tumors, especially GC.

### Supplementary Information

Supplementary file 1 (JPG 522 KB)—Fig. S1 Molecular characteristics of 10 *ARID1A*-mutant gastric cancer patients. Blue boxes indicate missense mutations, orange boxes are truncating mutations, red boxes are fusion, black boxes are deep deletion and brown boxes are MSS/MSI-L. GC, Gastric cancer; MSI, Microsatellite instability; PD-L1, Programmed cell death ligand-1; NA, Not appliable

Supplementary file 2 (XLSX 23 KB)

## Data Availability

All data generated during this study are included in this maunscript. The bioinformation analysis datasets generated in the current study are available online as cited in the materials and methods.
