# Supplementary figures and images for: Efficacy of immunotherapy in ARID1A-mutant solid tumors: a single-center retrospective study
Source: Discov Oncol. 2024 Jun 7;15:213. doi: 10.1007/s12672-024-01074-1 (PMC11161453; doi:10.1007/s12672-024-01074-1)

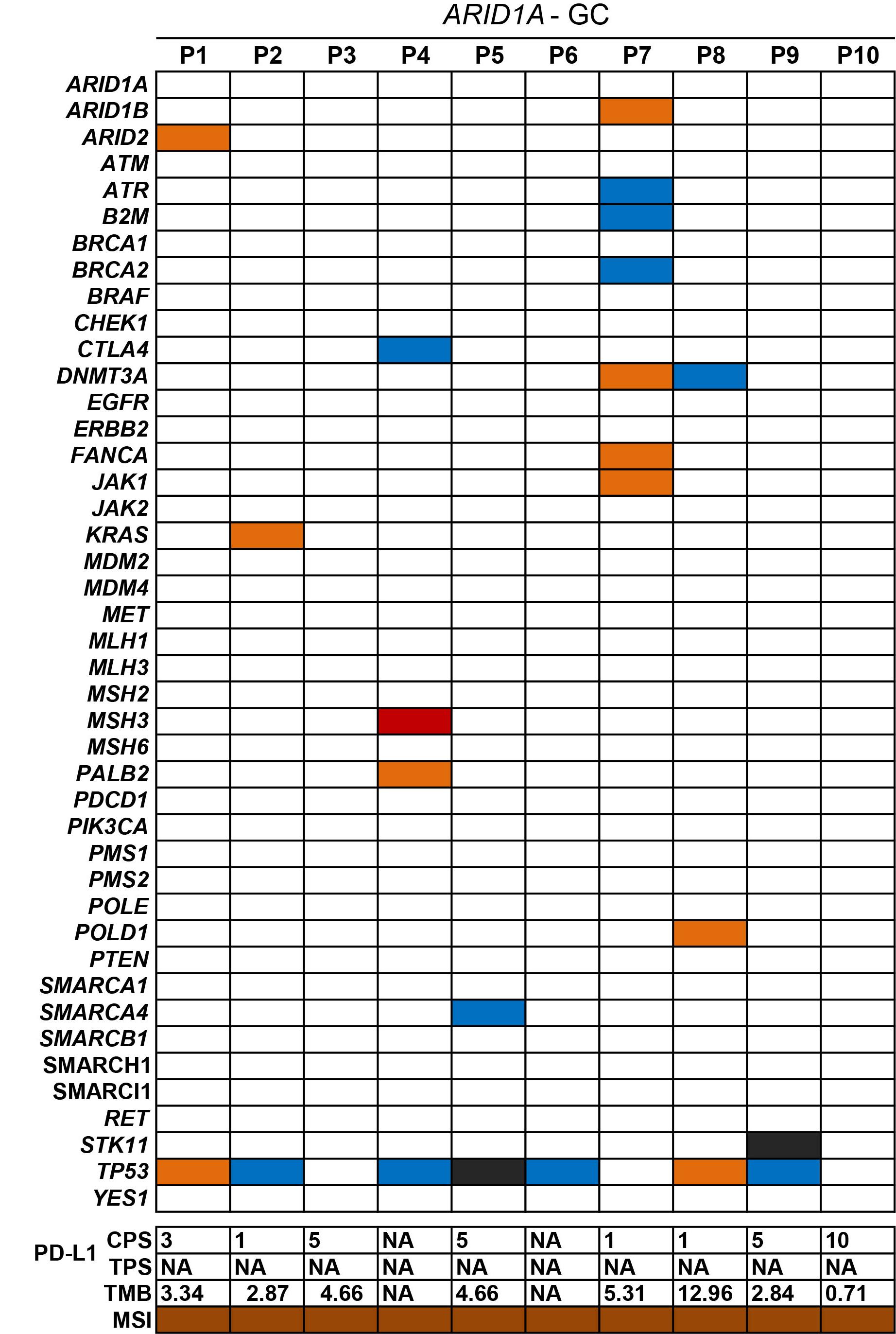

Supplement: Supplementary file 1 — (JPG 522 KB)—Fig. S1 Molecular characteristics of 10 ARID1A-mutant gastric cancer patients. Blue boxes indicate missense mutations, orange boxes are truncating mutations, red boxes are fusion, black boxes are deep deletion and brown boxes are MSS/MSI-L. GC, Gastric cancer; MSI, Microsatellite instability; PD-L1, Programmed cell death ligand-1; NA, Not appliable [file 12672_2024_1074_MOESM1_ESM.jpg]
